# The rare orange-red colored *Euphorbia pulcherrima* cultivar ‘Harvest Orange’ shows a nonsense mutation in a flavonoid 3’-hydroxylase allele expressed in the bracts

**DOI:** 10.1186/s12870-018-1424-0

**Published:** 2018-10-03

**Authors:** Daria Nitarska, Carmen Stefanini, Christian Haselmair-Gosch, Silvija Miosic, Benjamin Walliser, Maja Mikulic-Petkovsek, Ionela Regos, Ana Slatnar, Thomas Debener, Diro Terefe-Ayana, Vinicius Vilperte, Johannes Hadersdorfer, Karl Stich, Heidi Halbwirth

**Affiliations:** 1 Institute of Chemical, Environmental and Bioscience Engineering, Technische Universität Wien, 1060 Vienna, Austria; 2 Fruit Science, Technical University of Munich, 85354 Freising, Germany; 3 Agronomy Department, Fruit, Wine and Vegetable Growing, Biotechnical Faculty, University of Ljubljana, 1000 Ljubljana, Slovenia; 4 Institute of Plant Genetics, Leibniz Universität Hannover, 30419 Hannover, Germany

**Keywords:** Poinsettia (*Euphorbia pulcherrima*), Bract coloration, Flavonoid 3′-hydroxylase (F3′H), Dihydroflavonol 4-reductase (DFR), Substrate specificity, Anthocyanin, Pelargonidin, Cyanidin

## Abstract

**Background:**

Commercially available poinsettia (*Euphorbia pulcherrima*) varieties prevalently accumulate cyanidin derivatives and show intense red coloration. Orange-red bract color is less common. We investigated four cultivars displaying four different red hues with respect to selected enzymes and genes of the anthocyanin pathway, putatively determining the color hue.

**Results:**

Red hues correlated with anthocyanin composition and concentration and showed common dark red coloration in cultivars ‘Christmas Beauty’ and ‘Christmas Feeling’ where cyanidin derivatives were prevalent. In contrast, orange-red bract color is based on the prevalent presence of pelargonidin derivatives that comprised 85% of the total anthocyanin content in cv. ‘Premium Red’ and 96% in cv. ‘Harvest Orange’ (synonym: ‘Orange Spice’). cDNA clones of flavonoid 3*′*-hydroxylase (*F3′H*) and dihydroflavonol 4-reductase (*DFR*) were isolated from the four varieties, and functional activity and substrate specificity of the corresponding recombinant enzymes were studied. Kinetic studies demonstrated that poinsettia DFRs prefer dihydromyricetin and dihydroquercetin over dihydrokaempferol, and thus, favor the formation of cyanidin over pelargonidin. Whereas the *F3′H* cDNA clones of cultivars ‘Christmas Beauty’, ‘Christmas Feeling’, and ‘Premium Red’ encoded functionally active enzymes, the *F3′H* cDNA clone of cv. ‘Harvest Orange’ contained an insertion of 28 bases, which is partly a duplication of 20 bases found close to the insertion site. This causes a frameshift mutation with a premature stop codon after nucleotide 132 and, therefore, a non-functional enzyme. Heterozygosity of the *F3′H* was demonstrated in this cultivar, but only the mutated allele was expressed in the bracts. No correlation between *F3′H*-expression and the color hue could be observed in the four species.

**Conclusions:**

Rare orange-red poinsettia hues caused by pelargonidin based anthocyanins can be achieved by different mechanisms. F3′H is a critical step in the establishment of orange red poinsettia color. Although poinsettia DFR shows a low substrate specificity for dihydrokaempferol, sufficient precursor for pelargonidin formation is available *in planta,* in the absence of F3*’*H activity.

**Electronic supplementary material:**

The online version of this article (10.1186/s12870-018-1424-0) contains supplementary material, which is available to authorized users.

## Background

Poinsettia (*Euphorbia pulcherrima*) is a prominent ornamental plant of particular seasonal interest. The deep red coloration of their bracts induced by short days is typically associated with Christmas time in North America, Europe and Asia [1]. The bracts escort the relatively small and unimpressive reproductive structures and - as flowers - serve the function of attracting pollinators. Phylogenetically, they are leaves changing their function from photosynthesis providing assimilates for growth towards pollinator attraction [2–4]. Because of increasing competition and growing price pressure, more and more varieties are released. Breeding of poinsettia focuses on plant shape, shipping tolerability, robustness in cultivation, disease resistance, as well as on flowering time and showy color. Although the majority of cultivars show intense red bract coloration, other colors have become more popular in recent years such as pink, white, cinnamon and yellow or even bicolored, scattered or marbled types [1].

**Fig. 1:**
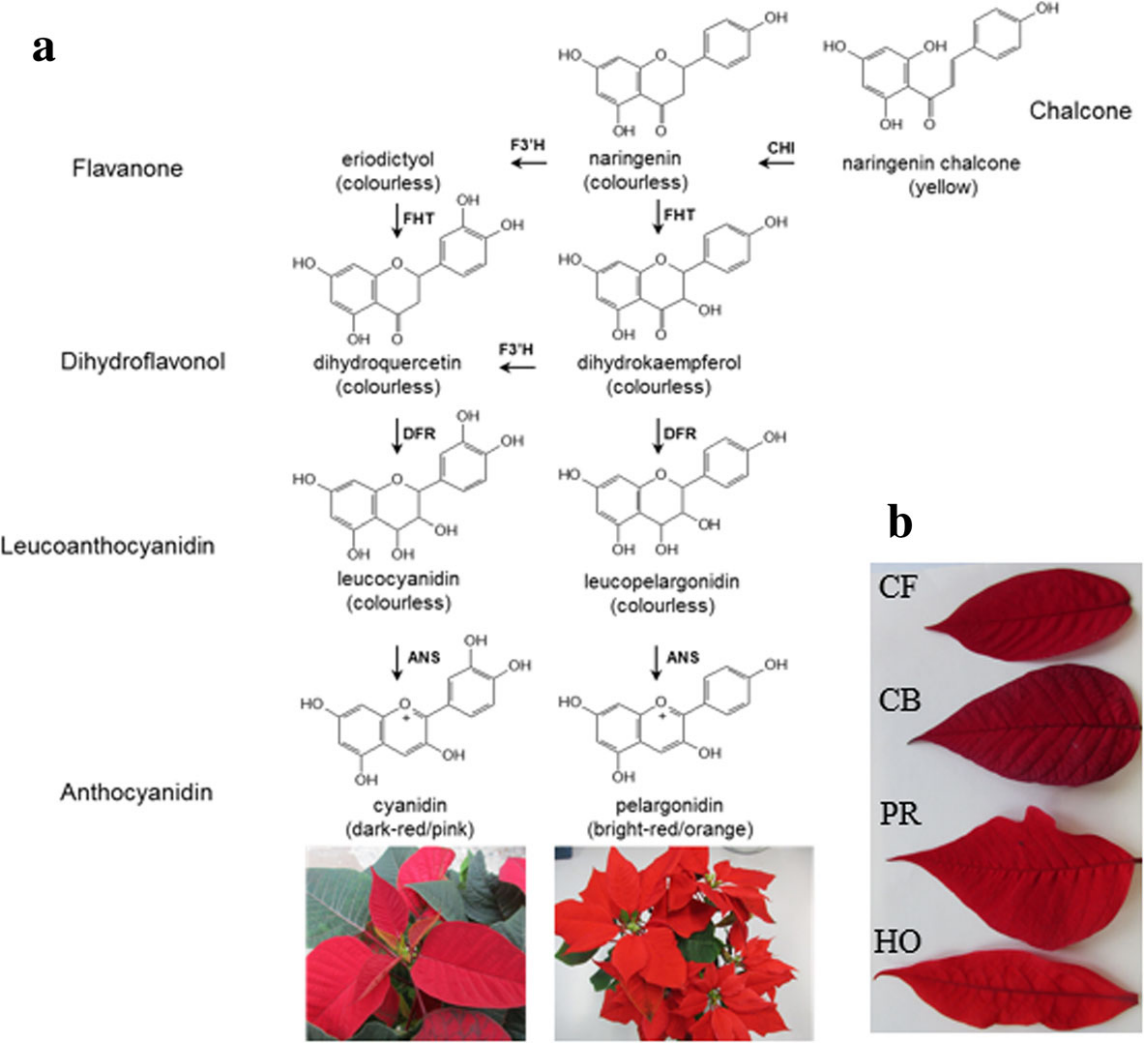
**a** Simplified overview of the anthocyanin pathway. Abbrev: ANS: anthocyanidin synthase, CHI: chalcone isomerase, CHS: chalcone synthase, DFR: dihydroflavonol 4-reductase, FHT: flavanone 3-hydroxylase, F3′H: flavonoid 3′-hydroxylase, F3′5′H: flavonoid 3′,5′-hydroxylase. **b** *Euphorbia pulcherrima* cv. ‘Christmas Feeling’ (CF), cv. ‘Christmas Beauty’ (CB), cv. ‘Premium Red’ (PR), cv. ‘Harvest Orange’ (HO)

Red colors of poinsettia bracts are caused by anthocyanins [5], which are widely distributed plant pigments in flowers, fruits and other plant tissues. Anthocyanins can impart the full spectrum of red hues to poinsettia bracts, from orange, red, rosy and, pink to crimson. In the most common red poinsettias, cyanidin type anthocyanins (two hydroxy groups in B-ring) are prevalent, but pelargonidin type anthocyanins (one hydroxy group in the B-ring) are also present to some extent [6] (Fig. 1a). Even traces of the delphinidin type pigments (three hydroxy groups in B-ring), have been previously found in poinsettia [6]. The hydroxylation pattern of the B-ring of the dihydroflavonol precursors ultimately determines the anthocyanin type that is accumulated. Dihydroflavonols with one hydroxy group (dihydrokaempferol, DHK) are the precursors for orange-red pigments (pelargonidin type), with two hydroxy groups (dihydroquercetin, DHQ) for red and pink pigments (cyanidin type) and with three hydroxy groups (dihydromyricetin, DHM) purple to blue pigments (delphinidin type).

The hydroxylation pattern of flavonoids and anthocyanins is determined by different enzymes (Fig. 1a). Flavonoid 3′ -hydroxylase (F3′H) and flavonoid 3′5’-hydroxylase (F3′5′H) are essential for the introduction of a second and third hydroxy group in the B-ring of flavonoids [7]. The F3′H (EC 1.14.13.21) belongs to the subfamily CYP75B of cytochrome P450-dependent monooxygenases (P450). This enzyme class is remarkably diverse and their members are present in all types of organisms [8, 9]. Plant P450s are usually membrane-bound enzymes associated with the endoplasmic reticulum [10]. The F3′H can accept flavanones and dihydroflavonols as well as leucoanthocyanidins (flavan 3,4-diols) as substrates, and can, therefore, influence the B-ring hydroxylation pattern at all precursor levels of anthocyanidin formation [11].

Another enzyme with impact on flower color is the dihydroflavonol 4-reductase (DFR, EC 1.1.1.219). The oxidoreductase catalyzes in the presence of NADPH the stereospecific reduction of the keto group in position 4 of dihydroflavonols, producing leucoanthocyanidins as precursors for anthocyanidin formation [12, 13]. The enzyme can show substrate specificity with respect to the B-ring hydroxylation pattern of the dihydroflavonol substrate and can, therefore, have an influence on the type of anthocyanin formed [14]. Flowers that accumulate pelargonidin type anthocyanins are usually characterized by low or absent F3′H and F3′5′H activities and possess a DFR that converts DHK (one hydroxy group in the B-ring) to a sufficient extent [15].

We studied the anthocyanin formation of two commonly dark-red cultivars (cvs. ‘Christmas Feelings’ and ‘Christmas Beauty’), and two orange-red cultivars (cvs. ‘Premium Red’ and ‘Harvest Orange’), (Fig. 1b). We show that the orange-red coloration of cv. ‘Harvest Orange’ is based on the almost exclusive accumulation of pelargonidin type pigments and that this correlates with a nonsense mutation in the *F3′H* gene, whereas in cv. ‘Premium Red’, which prevalently accumulates pelargonidin type anthocyanins, a functionally active F3′H is present. Our study establishes the base for designing strategies for breeding orange-red poinsettias accumulating prevalently pelargonidin type anthocyanin pigments.

## Methods

### Chemicals

(2-^14^C)-Malonyl-coenzyme A (55 mCi/mmol) was purchased from New England Nuclear Corp. GmbH (Vienna, Austria). (^14^C)-Labeled flavonoids naringenin, DHK, DHQ, and DHM were synthesized as previously described [16, 17] using recombinant F3’5’H from *Sollya heterophylla* and recombinant F3′H from *Arabidopsis thaliana*.

Pelargonidin-3-*O*-glucoside chloride, pelargonidin-3-*O*-rutinoside chloride, and, cyanidin-3-*O*-galactoside chloride available from Carbosynth (Berkshire, UK), cyanidin-3-*O*-glucoside was purchased from Extrasynthese (Genay, France) and cyanidin-3-*O*-rutinoside was obtained from Roth (Karlsruhe, Germany).

### Plant material

The analysis was carried out with young bracts of commercially available *Euphorbia pulcherrima* cv. ‘Premium Red’ (PR) (Dümmen Orange GmbH, Rheinsberg, Germany), cv. ‘Christmas Feelings’ (CF) and cv. ‘Christmas Beauty’ (CB) (Klemm + Sohn GmbH & Co. KG, Stuttgart, Germany), and cv. ‘Harvest Orange’ (HO) (Ecke Ranch, Encinitas, USA). After the takeover of Ecke Ranch by Dümmen Orange, cv. ‘Harvest Orange’ was sold as cv. ‘Orange Spice’. The plant material was collected in December 2015 and December 2016, frozen in liquid nitrogen and stored at − 80 °C. For HPLC analysis, samples were freeze-dried and ground in a ball mill.

### Analysis of anthocyanins

Anthocyanin extraction was performed by adding 500 μL of 5% acetic acid in methanol containing 3-methoxyflavone (0.02 mg/ml) as internal standard to 100 mg of powder for a period of 45 min in an ultrasonic water bath at 5 °C. After centrifugation (10,000 x g, 10 min, 4 °C), the clear supernatant was transferred to an Eppendorf tube. A 10 μL sample of the extract was injected for HPLC analysis. The anthocyanins were separated with an RP-HPLC system consisting of two pumps (model 422, Kontron Instruments, Germany), an automatic sample injector (model 231, Gilson Abimed Systems, Germany) and a diode array detector (Kontron 540, Kontron Instruments). Chromatography was performed on a Nucleosil column (250 × 4 mm, Macherey-Nagel, Germany) with a mobile phase consisting of water containing 5% formic acid (solvent A) and methanol (solvent B) with gradient elution (Additional file 1: Table S1). Anthocyanins were monitored and analyzed on their maximum UV-Vis absorption at 540 nm. Cyanidin-3-*O*-galactoside, cyanidin-3-*O*-glucoside, cyanidin-3-*O*-rutinoside, pelargonidin-3-*O*-glucoside and pelargonidin-3*-O*-rutinoside were available as authentic reference compounds. Quantification was performed using an internal standard method and calculating response factors for the standards at each concentration point on the calibration curve within the linear range. Linearity was measured at 5 concentrations. Calibration curves were constructed by plotting peak area versus concentration at 5 concentrations between 0.1–1 mg/mL for all reference compounds. Linearity was described by a regression equation and by the determination of the correlation coefficient. The identity of the anthocyanins was additionally confirmed by LC-MS analysis of cv. ‘Premium Red’ (Additional file 2: Table S2). LC-MS analysis was performed as previously described [6] using a mass spectrometer (LCQ Deca XP MAX, Thermo Scientific) with electrospray ionization (ESI) operating in positive ion mode using MS^2^ scanning mode from *m/z* 115 to 900.

### Enzyme preparation

Crude protein extracts from poinsettia bracts were obtained using protocol 1 as described earlier [18]. Briefly, 1 g bracts were homogenized with 0.5 g quartz sand and 0.5 g Polyclar AT with 6 ml 0.1 M KH_2_PO_4_/K_2_PO_4_ buffer (pH 6.5, containing 0.4% Na ascorbate). Low molecular compounds were removed by passing the crude protein extract preparation through a gel chromatography column (Sephadex G25, GE Healthcare, Freiburg, Germany). For *Euphorbia pulcherrima* DFR (*Ep*_DFR) enzyme characterization, enzyme preparation from commercially available red poinsettia was used.

### Enzyme assays

DFR assays with enzyme preparations from poinsettia bracts were performed using DHK, DHQ and DHM as substrates. The reaction contained in the final volume of 50 μL: 1–5 μL enzyme preparation, 0.048 nmol (^14^C)-dihydroflavonol, 0.25 nmol NADPH, and 40–44 μL 0.1 M KH_2_PO_4_/K_2_PO_4_ buffer (pH 6.5 for DHK; 6.25 for DHQ; 5.75 for DHM) containing 0.4% Na ascorbate. The amount of enzyme was set up to provide that the maximum conversion rate of the best substrate was around 50% (linear range of reaction). The reaction mixture with DHK and DHQ as a substrate was incubated at 40 °C for 20 min, and stopped and extracted with 70 μL of ethyl acetate. The organic phases were transferred to pre-coated thin-layer cellulose plates without fluorescence indication (Merck, Germany) and developed in chloroform/acetic acid/water (10:9:1, v:v:v). Assays with DHM as substrate were incubated at 40 °C for 20 min and stopped with 10 μL of 100% acetic acid and 30 μL of methanol. The mixture was chromatographed on 20 cm × 1 cm stripes of paper (Schleicher Schuell, 2041 b, Germany) in chloroform/acetic acid/water (10:9:1, v:v:v). Results were evaluated on a Berthold LB 2842 Linear Analyzer (Berthold, Germany) by integration of the peak areas.

For F3′H assays with crude protein preparations from bracts or recombinant enzymes obtained from yeast, the reaction contained in the final volume of 100 μl: 40 μL enzyme preparation (1 μg/μL enzyme), 0.048 nmol (^14^C)-naringenin or DHK, 0.05 nmol NADPH, and 55 μL 0.1 M KH_2_PO_4_/K_2_PO_4_ buffer pH 7.5 containing 0.4% Na ascorbate. The reaction mixture was incubated at 30 °C for 30 min and stopped with 10 μL 100% acetic acid. Substrate and product of the reaction were extracted with 70 μL ethyl acetate. The organic phases were transferred to pre-coated thin-layer cellulose plates without fluorescence indication (Merck, Germany) and developed in chloroform/acetic acid/water (10:9:1, v:v:v). Results were evaluated on a Berthold LB 2842 Linear Analyzer (Berthold, Germany) by integration of the peak areas.

Assays with enzyme preparations for chalcone synthase/chalcone isomerase (CHS/CHI), flavanone 3-hydroxylase (FHT) and flavonol synthase (FLS) were performed as described [18]. Separate detection of CHS and CHI is not possible because of the immediate chemical conversion of naringenin chalcone by CHI to naringenin without any cofactor requirements.

### Transcriptome analysis

De novo transcriptome assembly was performed using the bioinformatic tool Trinity v2.2.0 [19]. Homology searches and functional annotation were performed using Blast2GO v4.0 and the non-redundant protein sequence database of NCBI (ftp://ftp.ncbi.nlm.nih.gov/blast/db).

### Cloning of *F3′H*s

mRNA was extracted from poinsettia bracts with the μMACS mRNA isolation Kit (Miltenyi Biotec, Germany). cDNA was synthesized using the SuperScript II Reverse Transcriptase (Invitrogen, USA) and the primer oligo-dT SMART (AAGCAGTGGTATCAACGCAGAGTAC(T_23_)VN). Based on specific sequence information of *F3′H* fragments from an *E. pulcherrima* transcriptome study (Debener, unpublished), 5′-partial *F3′H* cDNA clones were isolated from the four poinsettia cultivars. The start codon was identified by alignment with the F3′H of the closely related species *Jatropha curcas* (Accession number XM_012224974). The 3′ end was identified by application of the 3′-RACE technique, using the SMARTer RACE 5′/3′ Kit (Clontech, Takara Bio Europe, France). Full size cDNA was amplified with the primer pair Ep_F3′H_full (Additional file 3: Table S3) using the *Taq/Pwo* Expand High Fidelity PCR System (Roche, Germany).

### Cloning of *DFR*s

Based on *DFR* sequences available in the NCBI database, the degenerated primer pair Ep-DFR1(deg) was designed (Additional file 3: Table S3). After amplification, *DFR* cDNA fragments were isolated, ligated in the vector pCR2.1-TOPO (Invitrogen, USA) and transformed into the *E. coli* strain TOP10. The obtained sequence information was used to design specific 3′- and 5′-RACE primers. Amplification of DFR 5′- and 3′-ends was performed using the SMARTer RACE 5′/3′ Kit (Clontech, Takara Bio Europe, France). The full size primer pair EpDFRfull was designed (Additional file 3: Table S3) and used for amplification of four full size *DFR*s from cv. ‘Christmas Beauty’, cv. ‘Christmas Feelings’, cv. ‘Premium Red’ and cv. ‘Harvest Orange’.

### Heterologous expression of *DFR* in *E. coli*

An established standard procedure for the production of soluble enzymes in *E. coli* was used for the heterologous expression of the DFR cDNA clone [20]. For each variety two PCR reactions with different primers were performed with *Pfu* DNA polymerase (Fermentas, Germany) (PCR1: *Ep*_*DFR*_LF and *Ep*_*DFR*_SR; PCR 2: *Ep*_*DFR*_SF and *Ep*_*DFR*_LR) (Additional file 3: Table S3). The PCR products were analyzed on agarose gel, eluted and purified. PCR products from both PCRs were mixed in an equimolar amount, denatured and reannealed, resulting in 1/4 double stranded DFR with sticky *Bam*HI (GATC) and *Eco*RI (AATT) recognition sequences at the ends for direct ligation into the linearized plasmid pGEX-6P-1 with T4 DNA ligase (Promega, Germany). After transformation into *E. coli* TOP10, plasmids were isolated and the presence of the insert confirmed by sequencing (Microsynth Austria AG, Austria). DFR sequences obtained during the present study were deposited in the NCBI database with the following accession numbers: KY273436 (*Ep*CB_DFR), KY273437 (*Ep*CF_DFR), KY499617 (*Ep*PR_DFR), KY273438 (*Ep*HO_DFR).

### Heterologous expression of *F3′H* in yeast

Heterologous expression of the *F3*’*H* cDNA clones, which encode membrane bound enzymes, was performed in the yeast *Saccharomyces cerevisiae* according to established procedures [21]. *F3*’*H* cDNA clones were amplified with the *Taq/Pwo* Expand High Fidelity PCR System (Roche, Germany), and ligated into the vector pYES2.1/V5-His-TOPO (Invitrogen, USA). Plasmids were isolated and the presence and sense orientation of the insert was confirmed by sequencing (Microsynth Austria AG, Austria). The vectors containing the *F3*′*H* cDNAs of the four cultivars were transformed into the yeast strain INVSc1 using the *Sc*. EasyComp Transformation Kit (Invitrogen, USA). Heterologous expression and preparation of protein fractions were carried out as described previously [21]. Protein fractions were shock frozen in liquid nitrogen and stored at − 80 °C.

### Phylogenetic analysis of F3′Hs

*F3′H* sequences obtained during the present study were deposited in the NCBI database with the following accession numbers: KY273439 (*Ep*CB*_*F3′H), KY273440 (*Ep*CF*_*F3′H)*,* KY489667 (*EpPR_*F3′H) and KY273441 (*Ep*HO*_*F3′H). Multiple alignments were carried out with the software MultAlin [22]. Amino acid sequences were aligned using MUSCLE [23]. The alignment was used for reconstruction of phylogenetic relationships on the JTT matrix-based model [24]. Initial trees for the heuristic search were obtained automatically by applying Neighbor-Join and BioNJ algorithms to a matrix of pairwise distances estimated using a JTT model. Evolutionary analyses were performed in MEGA7 [25]. Amino acid sequences used for this analysis were *Ep*CB*_*F3′H (KY273439), *Ep*CF*_*F3′H (KY273440)*, Ep*PR*_*F3′H (KY489667), *Ep*HO*_*F3′H (KY273441), *Arabidopsis thaliana* F3′H (AF271651), *Callistephus chinensis* F3′H (AF313488), *Gentiana triflora* F3′H (AB193313), *Gerbera hybrida* F3′H (ABA64468), *Glycine max* F3′H (AF499731), *Hieracium pilosella* F3′H (DQ319866), *Ipomoea nil* F3′H (AB113264), *Lobelia erinus* F3′H (BAF49324), *Matthiola incana* F3′H (AF313491), *Osteospermum hybrida* F3′H (ABB29899), *Pelargonium hortorum* F3′H (AF315465) *Perilla frutescens* F3′H (AB045593), *Petunia hybrida* F3′H (AF155332), *Torenia hybrida* F3′H AB057673, *Prunus avium* F3′H (ADZ54783), *Jatropha curcas* F3′H (XP_012080364), *Ricinus communis* F3′H (XP002514665), *Vitis vinifera* F3′H (ALP48438), *Camelina sativa* F3′H (XP_010491421), *Vaccinium ashei* F3′H (BAO58432). Flavone synthase (FNSII) sequences: *Glycine max* FNSII (ACV65037), *Medicago truncatula* FNSII (ABC86159), *Dahlia pinnata* FNSII (AGA17938).

### qPCR studies

The *F3′H* gene expression was evaluated by qPCR using the StepOnePlus system (Applied Biosystems, Germany) and the SybrGreenPCR Master Mix (Applied Biosystems, Austria) according to the manufacturer’s protocol. The analysis was performed in three independent replicates and the results were normalized to the two control genes, *actin* and *glyceraldehyde 3-phosphate dehydrogenase* (*GAPDH*). The relative expression ratio was calculated according to MW Pfaffl [26]. During the qPCR analysis primer pairs were used according to (Additional file 3: Table S3), to quantify the relative expression of *F3′H* (q*EpF3′H*) in comparison to the housekeeping genes *actin* (q*EpAct*) and *GAPDH* (q*EpGAPDH*). Product specificity was confirmed by analysis of melting curves and gel electrophoresis.

### Site-directed mutagenesis

Mutagenesis was performed by use of the Q5 Site-Directed Mutagenesis Kit (New England Biolabs, Austria) and the pGEX-6P-1 vector containing EpCF_DFR. Primers Ep_DFR_132L were designed with the NEBase Changer™ v 1.25 provided at http://nebasechanger.neb.com. The sequences of the primers are presented in (Additional file 3: Table S3). Success of mutation was confirmed by sequencing.

### Zygosity status of F3′H

The primer pair *Ep*F3′H_fra flanking the variable region at the N-terminal end of F3′H for all three varieties were designed (Additional file 3: Table S3). Gene fragments were amplified from genomic DNA, which was obtained according to Lipp et al. [27] using the *Taq/Pwo* Expend High Fidelity system (Roche, Germany). The expected band sizes were 107 (*Ep*CB_*F3′H*, and *Ep*PR_*F3′H*), 110 (*Ep*CF_*F3′H*), and 137 bp (*Ep*HO_*F3′H*) respectively. The PCR products were analyzed by electrophoresis in a 3% agarose gel and extracted with Wizard SV Gel and PCR Clean-up System (Promega, USA). After extraction from the gel, the PCR products were ligated into the vector pCR2.1-TOPO (Invitrogen, USA) and sequenced.

## Results

### Identification of anthocyanins

The anthocyanin contents and concentrations showed significant differences between cultivars exhibiting dark red bracts and the cultivars with orange-red bracts. Highest anthocyanin concentrations were found in cv. ‘Christmas Beauty’ (Table 1). The two dark red cultivars showed higher anthocyanin concentrations than the orange-red cultivars. The dark red cultivars contained cyanidin-3-*O-*glucoside, cyanidin-3-*O-*rutinoside, cyanidin-3-*O-*galactoside, pelargonidin-3-*O-*glucoside and pelargonidin-3-*O-*rutinoside (Table 1, Additional file 2: Table S2, Additional file 4: Figure S1) as reported earlier by Asen et al. [28], with cyanidin-3-*O-*glucoside and cyanidin-3-*O-*rutinoside as prevalent pigments. The orange-red cv. ‘Harvest Orange’ in contrast, produced only the two pelargonidin glycosides and in a few, but not all, biological replications, traces of cyanidin 3-*O*-glucoside (Table 1, Additional file 4: Figure S1). The orange-red cv. ‘Premium Red’ contained 82% pelargonidin glycosides and 18% cyanidin glycosides (Table 1). Pelargonidin-3-*O-*glucoside was the prevalent pigment in the orange-red cultivars.

**Table 1:** The anthocyanins in bracts of poinsettia cultivars and their respective concentration as determined by HPLC and LC-MS analysis of extracts

Anthocyanin composition [mg/g FW]	‘Christmas Beauty’	‘Christmas Feeling’	‘Harvest Orange’	‘Premium Red’
Total anthocyanins	29.7 ± 1.7	29.0 ± 0.5	15.4 ± 0.7	9.3 ± 3.9
Pelargonidin 3-*O*-glucoside	6.4 ± 0.4	5.2 ± 0.9	9.9 ± 0.1	3.8 ± 0.6
Pelargonidin 3-*O*-rutinoside	3.0 ± 0.2	2.9 ± 0.6	5.0 ± 0.6	2.0 ± 0.1
Other pelargonidin derivatives^a^	≥ 0.02	≥0.01	≥0.02	≥0.02
Total pelargonidin based anthocyanins	9.4 ± 0.6	8.2 ± 0.1	14.9 ± 0.2	7.9 ± 3.6
Cyanidin-3-*O*-glucoside	5.3 ± 0.2	5.0 ± 0.9	0.06 ± 0.1	0.4 ± 0.1
Cyanidin-3-*O*-rutinoside	9.9 ± 0.4	10.4 ± 0.2	n.d.	0.7 ± 0.1
Cyanidin-3-*O*-galactoside	5.0 ± 0.7	5.4 ± 0.7	n.d.	0.2 ± 0.03
Other cyanidin derivatives^b^	≥0.06	≥ 0.05	n.d.	≥0.01
Total cyanidin based anthocyanins	20.3 ± 1.4	20.9 ± 0.3	0.06 ± 0.1	1.3 ± 0.2
Total delphinidin based anthocyanins	≥0.01	≥0.01	n.d.	≥0.01

^a^Pelargonidin 3-*O*-(6”malonylglucoside), Pelargonidin 3-*O*-(6”malonyldihexosid) ^b^Cyanidin-3-*O*-xyloside

### Enzyme activities of the anthocyanin pathway

In the enzyme preparations obtained from intense red and orange-red poinsettia bracts, the activity of CHS/CHI, FHT, DFR, and F3′H, the key enzymes for anthocyanin formation, were measured (Additional file 5: Table S4.). The activity of the membrane F3′H could not be observed, maybe because of a loss of activity during destruction of the cell membranes [10].

Enzyme preparations from bracts converted all three types of dihydroflavonol substrates (Additional file 6: Table S4), DHK, DHQ and DHM. To study the substrate specificity of DFR, kinetic studies were performed with enzyme preparations obtained from bracts of cv. ‘Christmas Feelings’. DFR reactions were optimized for each substrate. Reaction time and protein concentration was chosen in a way that the maximum conversion rate for the best substrate did not reach more than 50%. The kinetic data demonstrated the substrate specificity of DFR for DHM and DHQ in comparison to DHK. The *k*_cat_/*K*_m_ values (Table 2) indicate that the best substrate for DFR is DHM, and that there is only low substrate specificity for DHK.

**Table 2:** Characterisation of DFR from enzyme preparations of cv. ‘Christmas Feeling’ bracts

Substrate	DHK	DHQ	DHM
pH optimum	6.50	6.25	5.75
Temperature optimum	40 °C	40 °C	40 °C
Time linearity [min]	20	20	20
Protein linearity [μg in assay]	2.6	0.2	0.2
Apparent *k*_*cat*_ [μmol/kg*s]	97	78	3105
Apparent *K*_*m*_ [μM]	14	0.9	29
*k*_*cat*_/*K*_*m*_ [l/s*kg]	7	86	107

### Cloning and characterization of *F3*’*Hs* from *Euphorbia*

**Fig. 2:**
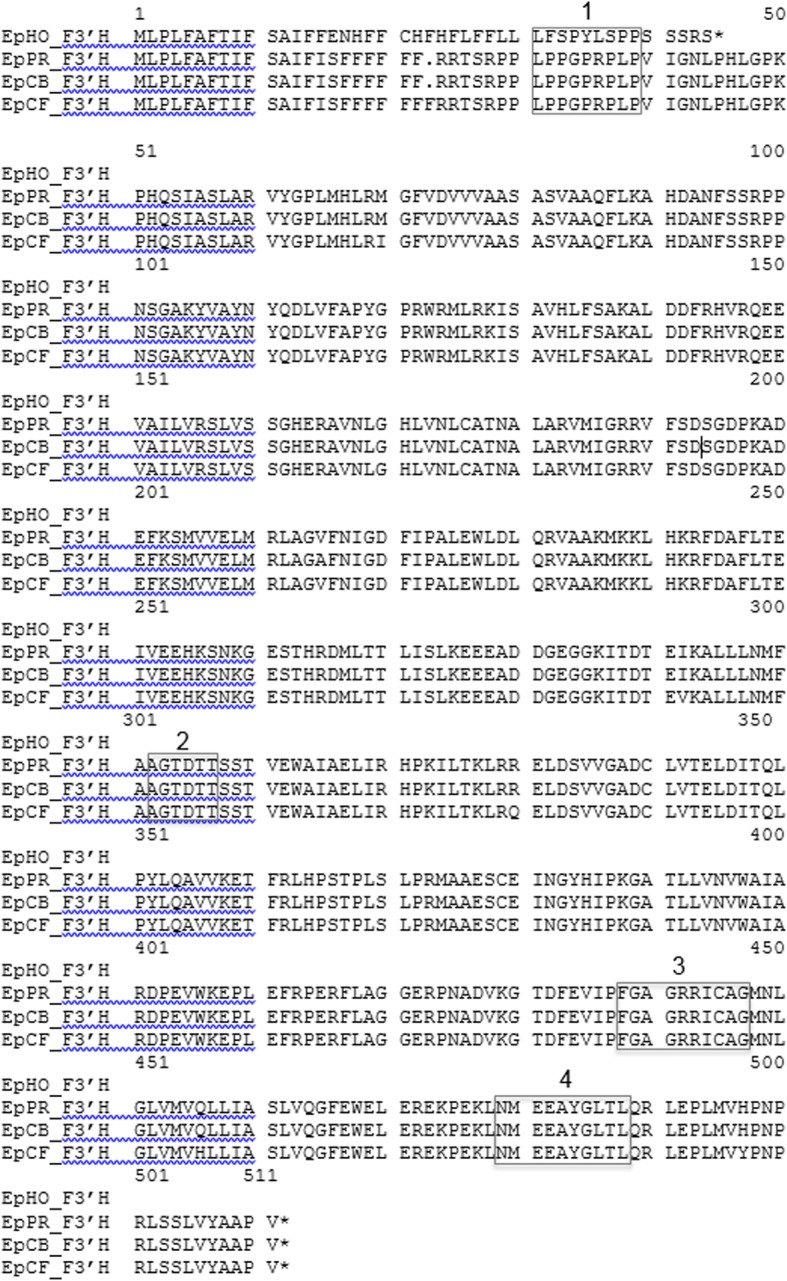
Multiple alignment of deduced amino acid sequences of *F3′H* cDNA clones of *Euphorbia pulcherrima* cvs. ‘Harvest Orange’ (*Ep*HO_*F3′H*, KY273441), ‘Premium Red’ (*Ep*PR_*F3′H*, KY489667), ‘Christmas Beauty’ (*Ep*CB_*F3′H*, KY273439), and ‘Christmas Feeling’ (*Ep*CF_*F3′H*, KY273440). Grey frames highlight characteristic sequences of the P450 protein family. 1. Proline-rich region [40]; 2. Oxygen binding pocket [41]; 3. Heme binding motif (Prosite pattern PS00086, [42]; 4. Substrate recognition site (SRS) 6 according to Seitz et al. [43]

Exploiting the preliminary data from a *Euphorbia* transcriptome study (Debener, unpublished) and the homology of the closely related species *Jatropha curcas,* four putative *F3*’*H* cDNA clones from poinsettias cvs. ‘Harvest Orange’, ‘Premium Red’, ‘Christmas Beauty’, and ‘Christmas Feelings’ were obtained (Accession numbers: KY273441, KY489667, KY273439, KY273440). The four cDNA clones showed 98.8% to 99.8% nucleotide sequence identities to each other (Additional file 6: Figure S2) and 67% to 76% to *F3*′*H* sequences from other species. The *F3′H* cDNA clones had open reading frames of 510 (*Ep*CB*_F3*′*H*, *Ep*PR*_F3*′*H*), 511 amino acids (*Ep*CF*_F3*′*H*), and 44 amino acids (*Ep*HO_F3′H), respectively (Fig. 2). The deduced *Ep*CF_*F3*′*H* amino acid sequence showed an additional phenylalanine in position 17 (numbering according to cv. ‘Christmas Feelings’) in the region responsible for anchoring the enzyme in the membrane [29].

**Fig. 3:**
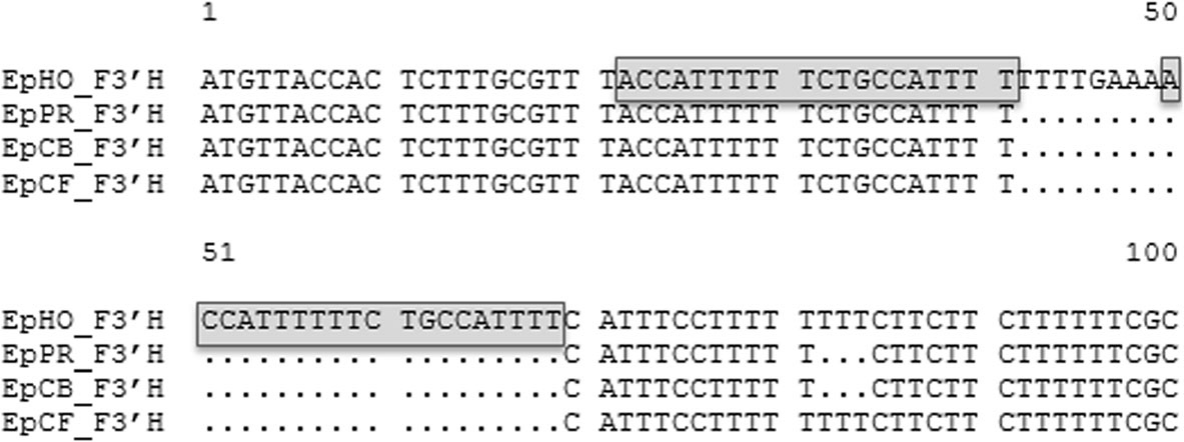
Multiple alignment of a selected part of the nucleotide sequences at the 5′-terminus of *F3′H* cDNA clones of *Euphorbia pulcherrima* cvs. ‘Harvest Orange’ (*Ep*HO_*F3′H*, KY273441), ‘Premium Red’ (*Ep*PR_*F3′H*, KY489667), ‘Christmas Beauty’ (*Ep*CB_*F3′H*, KY273439), and ‘Christmas Feeling’ (*Ep*CF_*F3′H*, KY273440). The grey-shaded frame highlights the repetition of ACCATTTTTTCTGCCATTTT from position 22 to 41 in position 50 to 69 (numbering from *Ep*HO_*F3′H*)

The nucleotide sequence of the *Ep*HO*_F3*′*H* cDNA clone was 31 bp longer compared to *Ep*CB*_F3*′*H, Ep*PR*_F3*′*H* and 28 bp longer compared to *Ep*CF*_F3*′*H* (Additional file 6: Figure S2). The *Ep*HO_*F3*′*H* nucleotide sequence carried an insertion of 28 bp in positions 42 to 69 (numbering according to cv. ‘Harvest Orange’). This included a stretch of 20 bp in positions 50–69, which is a repetition of the sequence 22ACCATTTTTTCTGCAATTTT41 (Fig. 3), and most importantly, results in a frameshift leading to an only 44 amino acids truncated F3′H fragment (Fig. 2).

The phylogenetic relationship of the poinsettia F3′Hs in comparison to F3′Hs from a further 23 species was analyzed using FNSII as outgroup. The deduced poinsettia F3′H amino acid sequences clustered together and showed closest relationship to putative F3′H sequences of *Ricinus communis* and *Jatropha curcas* (Additional file 7: Figure S3), which also belong to the same family Euphorbiaceae.

The cDNA clones were transferred into the pYES2.1/V5-His-TOPO vector and heterologously expressed in yeast. The recombinant enzymes *Ep*CB_F3′H, *Ep*CF_F3′H, and *Ep*PR_F3′H were functionally active and catalysed the NADPH dependent conversion of both naringenin and DHK to eriodictyol and DHQ, respectively. Both substrates were accepted to a comparable extent (Table 3). As expected, no activity of *Ep*HO_F3′H was observed (Table 3).

**Table 3:** Functional activity test with recombinant enzymes from *Euphorbia pulcherrima*

	DFR (DHK/DHQ/DHM)	F3′H (NAR/DHK)
Cultivar	nmol/s^a^kg	nmol/s^a^kg
‘Harvest Orange’	804/1260/800	0/0
‘Premium Red’	1560/2043/1960	424/345
‘Christmas Beauty’	200/2040/1987	420/370
‘Christmas Feeling’	1300/1870/1630	430/345
	0/0/187^a^	

^a^*Ep*CF_DFR132L mutant

### qPCR studies

**Fig. 4:**
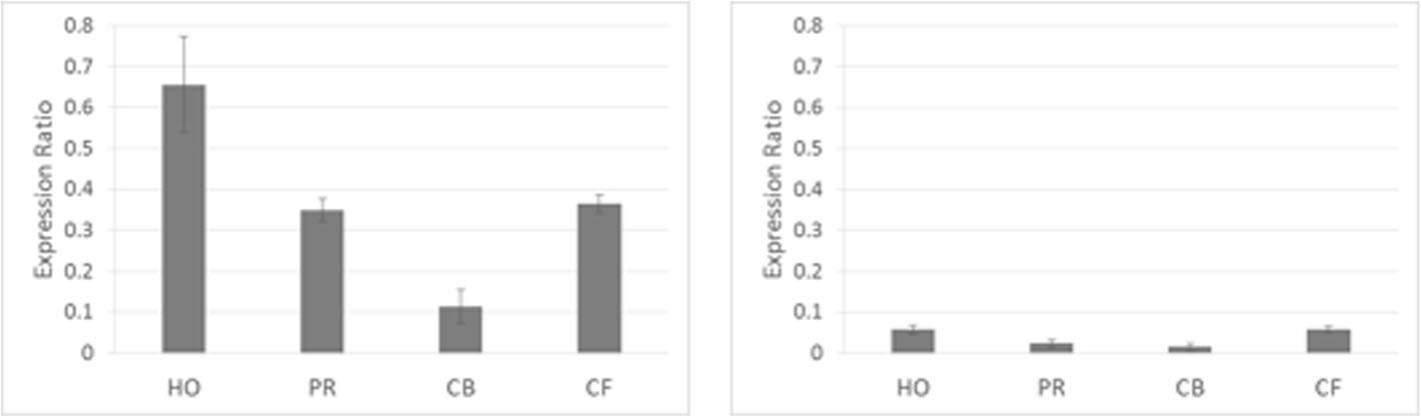
Quantitative expression of *F3′H* normalized to glycerine aldehyde 3-phosphate dehydrogenase (GAPDH) in *Euphorbia pulcherrima* cvs. ‘Harvest Orange’ (HO), ‘Premium Red’ (PR), ‘Christmas Beauty’ (CB), and ‘Christmas Feeling’ (CF). Left: Three year old plants kept in the greenhouse. Right: Plants in their first year cultivated in house under standard conditions. Data were calculated from three biological replicates with at least two technical replicates and with error bars representing standard deviation

The expression profile of *F3′H* was evaluated in the four poinsettia varieties using two sets of plants of different age and kept at different conditions. The quantitative real-time PCR data for *F3′H* were normalized against *glyceraldehyde 3-phosphate dehydrogenase* (*GAPDH,* Fig. 4) and *actin* (data not shown). Results obtained from both housekeeping genes were comparable. Generally higher expression ratios were observed for the 3 year old plants kept in the greenhouse which could be owing to the better light conditions. In both cases, however, there was no correlation between *F3′H* expression and cyanidin formation. Highest expression rates were found in the orange-red cv. ‘Harvest Orange’ whereas the dark red cv. ‘Christmas Beauty’ showed the lowest *F3′H* expression (Fig. 4).

### Zygosity status

**Fig. 5:**
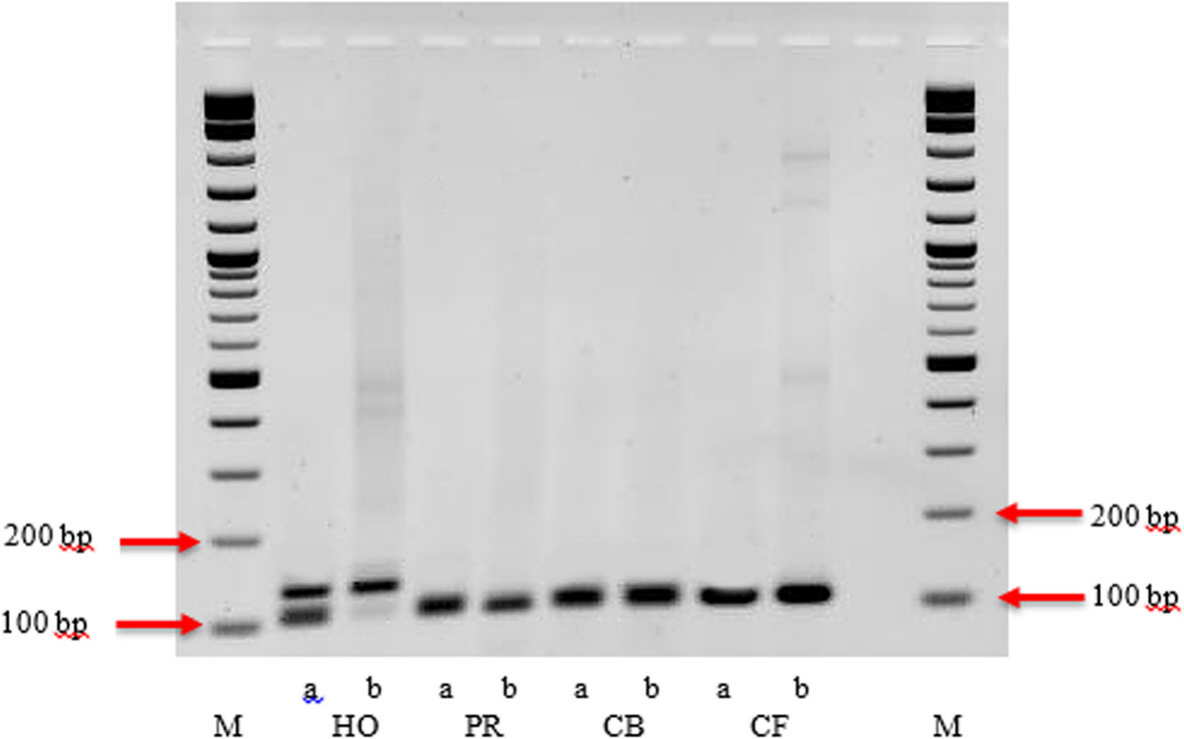
Amplification of *F3′H* with the primer pair *Ep*F3′H_fraF and *Ep*F3′H_fraR (Additional file 5: Table S4) flanking the variable region at the N-terminal end using genomic DNA (a) and cDNA (b) from the four poinsettia cultivars ‘Harvest Orange’ (HO), ‘Premium Red’ (PR), Christmas (CB) and ‘Christmas Feeling’ (CF). For cv. ‘Harvest Orange’, amplification from gDNA delivered two fragments of the expected size (calculated values: 109 and 138 bp), whereas only the larger fragment was obtained with cDNA. With gDNA and cDNA from the other cultivars only a single fragment of the smaller size was obtained. Size marker (M) was the 2-Log DNA Ladder (New England Biolabs, UK) with digested DNA fragments ranging from 100 bp to 10 kbp (100 bp steps between 100 and 1000); 100 and 200 bp fragments are highlighted on the gel with red arrows

During the isolation of the *F3′H* cDNA clones of the four cultivars, electropherograms provided by the sequencing company did not indicate that more than one version of *F3′H* is present. To confirm this and particularly to check if *Ep*HO_*F3′H* possesses another allele with a correct open reading frame, we designed primers (Additional file 3: Table S3) flanking the inserted region and performed PCR with genomic DNA and cDNA as template. Based on the isolated cDNA clones, we expected band sizes of 107 for *F3′H*s from cvs. ‘Christmas Beauty’ and ‘Premium Red’ and of 110 for the *F3′H*s from ‘Christmas Feelings’, respectively. For ‘Harvest Orange’, a size of 137 bp was expected in the case of a fragment carrying the insertion, whereas the presence of an allele without the insertion mutation would be indicated by a 109 bp amplicon. After the separation of the obtained amplicons on a 3% agarose gel, in the cultivars ‘Christmas Beauty’, ‘Christmas Feeling’ and ‘Premium Red’, only one band was present and the sizes corresponded to the expected fragment sizes (Fig. 5). For cv. ‘Harvest Orange’, the situation was different. When genomic DNA was used as a template, two bands of slightly different size were observed, of which, however, only one was expressed in the bracts. With cDNA as a template only the larger band, carrying the insert resulting in a frameshift, was obtained, whereas the smaller band was almost not visible (Fig. 5). Sequencing of the two fragments confirmed that in cv. ‘Harvest Orange’ two alleles of the *F3*′*H* gene are present of which one carries the insert mutation provoking the premature stop codon.

### Cloning and characterization of *DFR*s from poinsettia

Putative *DFR* cDNA clones were isolated from the bracts of cvs. ‘Christmas Beauty’, ‘Christmas Feelings’, ‘Premium Red’ and ‘Harvest Orange’. All four consisted of 1056 nucleotides, with open reading frames of 352 deduced amino acids (Additional file 8: Figure S4). The *DFR* cDNA clones showed a very high sequence identity between 98.3 and 99.4% on the amino acid level, even across the four independent varieties. During the isolation and characterization, a second allelic variant has not been isolated.

The *DFR* cDNA clones from cvs. ‘Harvest Orange’ (KY273438), ‘Premium Red’ (KY499617), ‘Christmas Feeling’ (KY273437), and ‘Christmas Beauty’ (KY273436) were cloned into the pGEX-6P-1 vector and heterologously expressed in *E. coli*. All four recombinant proteins were active, catalyzing the NADPH dependent conversion of dihydroflavonols to leucoanthocyanidins. They accepted DHQ and DHM as a substrate to a comparable extent (Table 3). DHK was accepted by recombinant *Ep*CF_DFR, *Ep*HO_DFR and *Ep*PR_DFR, whereas recombinant *Ep*CB_DFR showed only a low conversion rate of DHK (Table 3).

Substitution of the valine in position 132 (numbering from *Ep*CF_DFR) of *Ep*CF_DFR to leucine was performed, to change the VDV motif (Additional file 8: Figure S4) of the poinsettia DFR into the LDV motif commonly found in e.g. the petunia DFR (AAF60298) [14]. This resulted in an increase of DHM specificity but also in a major loss of enzyme activity (Table 3).

## Discussion

Anthocyanins are most frequently found in flowers and fruits, where they serve as colorful signals to pollinators and seed dispersers [30]. However, other tissues such as, leaves, roots, stems and shoots can accumulate anthocyanins as well. In the latter case, the function of anthocyanins is less well understood, but has repeatedly been shown to be involved in the protection against biotic and abiotic stress [31]. Anthocyanins in leaves have been suggested to fulfil a range of functions including screening against sun and UV-B radiation, antioxidative protection, osmoregulation and herbivory and pathogen defence [32].

Cyanidin, which carries 2 hydroxy groups in the B-ring, is regarded to be the ancestral pigment. Formation of pelargonidin and delphinidin, which carry 1 and 3 hydroxy groups, respectively, evolved in flowers by loss-of function mutations and gain of function mutations, respectively, as an adaptation to the colour sense of specific pollinators. Thus, cyanidin based anthocyanins are predominant in less advanced tissues such as leaves [33]. As bracts are specialized leaves associated with reproductive structures, it does not seem to be surprising that an intense red coloration prevalently based on cyanidin derivatives seems to be the standard within the huge spectrum of available commercial varieties of red poinsettia [5, 6, 28, 34]. Orange-red hues seem to be a rare occurrence in poinsettia and not to be simply the result of a specific selection of breeders for intense, dark-red colour hues. In this study, we analysed the anthocyanin content and the correlating enzyme activities and gene expressions of four poinsettia cultivars to identify possible mechanisms leading to orange-red bract coloration.

Recently DFR was suggested to take a key role in the conversion of green leaves into red bracts in poinsettia [35]. In addition, the formation of cyanidin type anthocyanins strongly depends on the presence of F3′H hydroxylating enzymes, but can also be influenced by DFR substrate specificity [16]. Our studies therefore concentrated on these two enzymes.

The orange-red cvs. ‘Harvest Orange’ and ‘Premium Red’ were characterized by a generally lower anthocyanin concentration and a prevalent presence of pelargonidin-type anthocyanins. The lower amounts of total anthocyanins present in the orange-red bracts correlated well with the observed low specificity of DFR for DHK, which could result in a lower total conversion rate of dihydroflavonols, if only DHK is present. The bright orange-red coloration of the cvs. ‘Harvest Orange’ and ‘Premium Red’ demonstrate however, that sufficient precursors for pelargonidin formation can be provided by poinsettia DFR despite its low substrate specificity for DHK. Similar observations were reported for carnations where both pelargonidin and cyanidin based phenotypes can be formed, despite a strong preference of DFR for DHQ and DHM in comparison to DHK [36]. A comparable situation was recently reported for petunia DFR [37]. Substrate specificity of DFR was reported to be determined in a highly variable region of 26 amino acids in the N-terminal part of the enzyme, apparently with particular relevance of amino acid 133 [14]. The DFRs of the four varieties showed high homology in this area and there was no indication of the presence of an allelic variant of the DFR in contrast to F3′H. All cDNA clones identified showed high activity and concordant substrate specificity. The preference for DHQ over DHK, if both are simultaneously present, could well explain the prevalence of cyanidin and also indicates that F3′H is the key enzyme in the formation of orange-red color in poinsettia as described earlier for other species [38].

For *F3′H*, in contrast, we were able to show the presence of two allelic variants, of which only one was expressed in the petals. The isolated full-size *F3′H* cDNA clones of cvs. ‘Christmas Beauty’, ‘Christmas Feeling’ and ‘Premium Red’ encoded functionally active enzymes with very few differences in their deduced amino acid sequences. The cDNA clone obtained from cv. ‘Harvest Orange’ had an insertion of 28 bases, which causes a frame shift and an early termination of the translation at amino acid 44, and, consequently, a non- functional F3′H, as demonstrated by heterologous expression in yeast. The insertion is, however, only present in the allele, which is actually expressed in the bracts. Expression of the other allele, which encodes presumably a functionally active F3′H without the insert mutation, was almost negligible. This provides a sufficient explanation for the almost exclusive presence of pelargonidin-type anthocyanins and the orange-red coloration in cv. ‘Harvest Orange’. The 20 nucleotide repetition in the insertion indicated that the frameshift mutation could have been caused by a transposition event [39]. It is possible that, as a result of transposition, a part of the sequence was repeated and one additional nucleotide remained after retransposition.

Quantification of the *F3′H* gene expression by real-time PCR in the four cultivars did not indicate any correlation with the color type. Lowest *F3′H* expression was measured for the prevalently cyanidin type anthocyanins containing cv. ‘Christmas Beauty’. The relatively high *F3′H* expression in the orange-red cv. ‘Premium Red’ was surprising because *Ep*PR_*F3′H* cDNA encoded a functionally active enzyme. At this stage it remains open if a post-transcriptional or a post-translational event or a simple competition between enzymes is responsible for the prevalence of pelargonidin derivatives formed in this cultivar.

## Conclusion

In bracts, anthocyanins serve the same purpose as in flowers, i.e. attraction of pollinators and their biosynthesis follows similar mechanisms as numerously reported for flowers [2]. Our studies have shown that the red hues of poinsettias are primarily influenced by the anthocyanin composition and that attractive orange-red color of poinsettia bracts essentially depends on the absence of cyanidin formation, which can be obtained by different mechanisms. An F3′H knock-out via a nonsense mutation could therefore be a promising approach for breeding orange-red poinsettia bracts by molecular breeding techniques. Future work will concentrate on application of these findings in molecular breeding approaches.

## Additional files

Additional file 1: **Table S1.** Gradient elution time-table program in the RP-HPLC system using mobile phase A (water with 5% HCO_2_H) and mobile phase B (MeOH). (DOCX 17 kb)

Additional file 2: **Table S2.** Identification of anthocyanins in poinsettia flowers by using their HPLC–DAD, LC–MS and LC–MS/MS data in the positive ion mode. (DOCX 16 kb)

Additional file 3: **Table S3.** List of primers used. (DOCX 16 kb)

Additional file 4: **Figure S1.** High performance liquid chromatographic profile of anthocyanins in (a) cv. Christmas Beauty and (b) cv. Harvest Orange. The anthocyanins in order of increasing retention time were cyanidin-3-*O*-galactoside (A1), cyanidin-3-*O*-glucoside (A2), pelargonidin-3-*O*-glucoside (A3), cyanidin-3-*O*-rutinoside (A4) and pelargonidin-3-*O*-rutinoside (A5). (DOCX 109 kb)

Additional file 5: **Table S4.** Activities of 3 key enzymes of the anthocyanin pathway in *Euphorbia pulcherrima. (DOCX 16 kb)*

Additional file 6: **Figure S2.** Multiple alignment of the open reading frames of the F3′H cDNA clones of *Euphorbia pulcherrima* cvs. Harvest Orange (EpHO_F3′H, KY273441), Premium Red (EpPR_F3′H, KY489667), Christmas Beauty (EpCB_F3′H, KY273439), and Christmas Feelings (EpCF_F3′H, KY273440). (DOCX 23 kb)

Additional file 7: **Figure S3.** Phylogenetic analysis of F3′Hs from the three poinsettia cvs. Christmas Feelings (KY273440), Christmas Beauty (KY273439) and Premium Red (KY489667) by application of the Maximum Likehood method based on the deduced amino acid sequences of isolated poinsettia F3′H cDNA clones and deduced F3′H amino acid sequences of other species available in the NCBI database. Sequences of FNSII were used as outgroup. The JJT matrix-based model was used as a substitution model. The percentage of trees in which the associated taxa clustered together is shown next to the branches. (DOCX 20 kb)

Additional file 8: **Figure S4.** Multiple Alignment of the deduced amino acid sequences of the DFRs of *Euphorbia pulcherrima* cvs. Harvest Orange (EpHO_DFR, KY273438), Premium Red (EpPR_DFR, KY499617), Christmas Beauty (EpCB_DFR, KY273436), and Christmas Feelings (EpCF_DFR, KY273437). Grey frames highlight the VDV region in position 132 to 134. Grey shades highlight differences in the amino acid sequence. (DOCX 21 kb)

## Abbreviations

CB: ‘Christmas Beauty’
CF: ‘Christmas Feeling’
CHS/CHI: Chalcone synthase/chalcone isomerase
cv(s): Cultivar(s)
DFR: Dihydroflavonol 4-reductase
DHK: Dihydrokaempferol
DHM: Dihydromyricetin
DHQ: Dihydroquercetin
F3’5’H: Flavonoid 3′,5′-hydroxylase
F3′H: Flavonoid 3′-hydroxylase
FHT: Flavanone 3-hydroxylase
FNS: Flavone synthase
gDNA: Genomic DNA
HO: ‘Harvest Orange’
PR: ‘Premium Red’
