# Supplementary material for: The rare orange-red colored Euphorbia pulcherrima cultivar ‘Harvest Orange’ shows a nonsense mutation in a flavonoid 3’-hydroxylase allele expressed in the bracts
Source: BMC Plant Biol. 2018 Oct 3;18:216. doi: 10.1186/s12870-018-1424-0 (PMC6171185; doi:10.1186/s12870-018-1424-0)
Supplement: Supplementary file 1 — Table S1. Gradient elution time-table program in the RP-HPLC system using mobile phase A (water with 5% HCO2H) and mobile phase B (MeOH). (DOCX 17 kb) [file 12870_2018_1424_MOESM1_ESM.docx]

**Table S1:** Gradient elution time-table program in the RP-HPLC system using mobile phase A (water with 5 % HCO_2_H) and mobile phase B (MeOH)

| **Time (min)** | **Flow (ml/min)** | **%A** | **%B** |
| --- | --- | --- | --- |
| 0-5 | 0.5 | 95 | 5 |
| 5-10 | 0.5 | 95-90 | 5-10 |
| 10-15 | 0.5 | 90 | 10 |
| 15-35 | 0.5 | 90-85 | 10-15 |
| 35-55 | 0.5 | 85 | 15 |
| 55-70 | 0.5 | 85-80 | 15-20 |
| 70-80 | 0.5 | 80 | 20 |
| 80-95 | 0.5 | 80-75 | 20-25 |
| 95-125 | 0.5 | 75-70 | 25-30 |
| 125-145 | 0.5 | 70-60 | 30-40 |
| 145-160 | 0.5 | 60-50 | 40-50 |
| 160-175 | 0.5 | 50-10 | 50-90 |
| 175-195 | 0.5 | 10 | 90 |
