# Supplementary material for: The rare orange-red colored Euphorbia pulcherrima cultivar ‘Harvest Orange’ shows a nonsense mutation in a flavonoid 3’-hydroxylase allele expressed in the bracts
Source: BMC Plant Biol. 2018 Oct 3;18:216. doi: 10.1186/s12870-018-1424-0 (PMC6171185; doi:10.1186/s12870-018-1424-0)
Supplement: Supplementary file 2 — Table S2. Identification of anthocyanins in poinsettia flowers by using their HPLC–DAD, LC–MS and LC–MS/MS data in the positive ion mode. (DOCX 16 kb) [file 12870_2018_1424_MOESM2_ESM.docx]

**Table S2:** Identification of anthocyanins in poinsettia flowers by using their HPLC–DAD, LC–MS and LC–MS/MS data in the positive ion mode.

| Anthocyanin | [M^+^] (*m/z*) | MS^2^ [M^+^] (*m/z*) |
| --- | --- | --- |
| Cyanidin 3-*O*-galactoside | 449 | 287 |
| Cyanidin 3-*O*-glucoside | 449 | 287 |
| Cyanidin 3-*O*-rutinoside | 595 | 449/287 |
| Pelargonidin 3-*O*-glucoside | 433 | 271 |
| Pelargonidin 3-*O*-rutinoside | 579 | 433/271 |
| Cyanidin 3-*O*-xyloside | 419 | 287 |
| Pelargonidin 3-*O*-(6"malonylglucoside) | 519 | 433/271 |
| Cyanidin 3-*O*-(6"malonylglucoside)-5-*O-*glucoside | 697 | 535/449/287 |
| Delphinidin 3-*O*-(2G-xylosylrutinoside) | 743 | 597/435/303 |
