# Supplementary material for: The rare orange-red colored Euphorbia pulcherrima cultivar ‘Harvest Orange’ shows a nonsense mutation in a flavonoid 3’-hydroxylase allele expressed in the bracts
Source: BMC Plant Biol. 2018 Oct 3;18:216. doi: 10.1186/s12870-018-1424-0 (PMC6171185; doi:10.1186/s12870-018-1424-0)
Supplement: Supplementary file 3 — Table S3. List of primers used. (DOCX 16 kb) [file 12870_2018_1424_MOESM3_ESM.docx]

**Table S3:** List of primers used

| Primer | Forward 5'-3' (F) | Reverse 5'-3' (R) |
| --- | --- | --- |
| Ep_F3'H_full | GCCAACTACATCTAATCTAACCCAACATG | GACAAACAGTACTGGAGTAATAAGCAACG |
| Ep_DFR1(deg) | GG(AGCT)TT(CT)AT(ACT)GG(GCT)TC(AT)TGGCT(CT)(AG)TCATGA | TC(AGT)A(CT)(AGCT)GC(ACT)CC(ACT)(AGCT)(CT)(AG)(AT)ACAT(AG)TCCTC |
| Ep_DFR_full | TGGGGAAGTTNTCTTGTTTTCA | TCCCATTATTTTTATTGTCTCCCTAGGTA |
| Ep_DFR_L | GATCCATGGGTGAAGTGCCTGAGATTGTG | AATTCTCCCATTATTTTTATTGTCTCCCTAGGTA |
| Ep_DFR_S | CATGGGTGAAGTGCCTGAGATTGTG | CTCCCATTATTTTTATTGTCTCCCTAGGTA |
| Ep_DFR_132L | GGCTGGCACACTTGATGTTGAG | GAGGATGTGAATATGATCC |
| qEpGAPDH | GTCAAGCAGGCTTCTCACTT | GCCAACCGGCTTGACAT |
| qEpAct | GCTCAGTCCAAGAGAGGTATTT | CCATGTCATCCCAATTGCTTAC |
| qEpF3'H | ACAGGAAGAAGTGGCAATCC | TCTTCTGCCAATCATTACGC |
| EpF3'H_fra | GCCAACTACATCTAATCTAACCCAACATG | GCGAGAGGTACGGCGAA |
