# Supplementary material for: The rare orange-red colored Euphorbia pulcherrima cultivar ‘Harvest Orange’ shows a nonsense mutation in a flavonoid 3’-hydroxylase allele expressed in the bracts
Source: BMC Plant Biol. 2018 Oct 3;18:216. doi: 10.1186/s12870-018-1424-0 (PMC6171185; doi:10.1186/s12870-018-1424-0)
Supplement: Supplementary file 4 — Figure S1. High performance liquid chromatographic profile of anthocyanins in (a) cv. Christmas Beauty and (b) cv. Harvest Orange. The anthocyanins in order of increasing retention time were cyanidin-3-O-galactoside (A1), cyanidin-3-O-glucoside (A2), pelargonidin-3-O-glucoside (A3), cyanidin-3-O-rutinoside (A4) and pelargonidin-3-O-rutinoside (A5). (DOCX 109 kb) [file 12870_2018_1424_MOESM4_ESM.docx]

**Figure S1:** High performance liquid chromatographic profile of anthocyanins in (a) cv. Christmas Beauty and (b) cv. Harvest Orange. The anthocyanins in order of increasing retention time were cyanidin-3-*O-*galactoside (A1), cyanidin-3-*O-*glucoside (A2), pelargonidin-3-*O-*glucoside (A3), cyanidin-3-*O-*rutinoside (A4) and pelargonidin-3-*O-*rutinoside (A5)

**
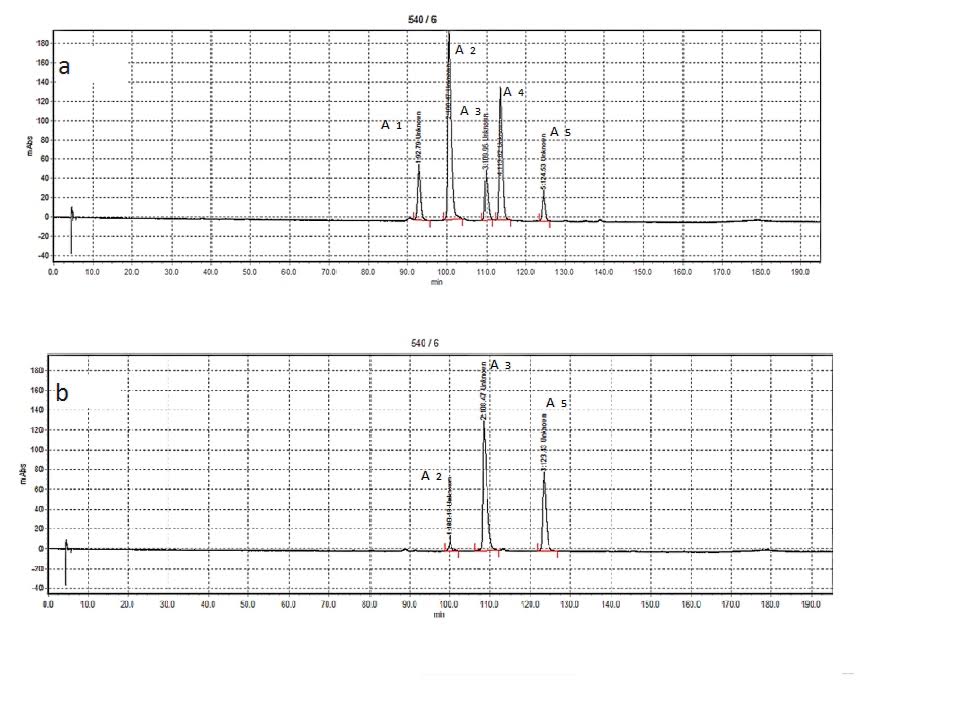
**
