# Supplementary material for: The rare orange-red colored Euphorbia pulcherrima cultivar ‘Harvest Orange’ shows a nonsense mutation in a flavonoid 3’-hydroxylase allele expressed in the bracts
Source: BMC Plant Biol. 2018 Oct 3;18:216. doi: 10.1186/s12870-018-1424-0 (PMC6171185; doi:10.1186/s12870-018-1424-0)
Supplement: Supplementary file 5 — Table S4. Activities of 3 key enzymes of the anthocyanin pathway in Euphorbia pulcherrima. (DOCX 16 kb) [file 12870_2018_1424_MOESM5_ESM.docx]

**Table S4:** Activities of 3 key enzymes of the anthocyanin pathway in *Euphorbia pulcherrima*

|  | **CHS/CHI** | **FHT** | **DFR** (DHK/DHQ/DHM) | |
| --- | --- | --- | --- | --- |
| **Cultivar** | nmol/s*g | nmol/s*g | | nmol/s*g |
| Harvest Orange | 0.9 | 1.2 | | 4.3/4.4/4.4 |
| Premium Red | 0.3 | 0.3 | | 1.9/4.5/4.4 |
| Christmas Feelings | 0.6 | 1.0 | | 4.2/4.3/4.5 |
| Christmas Beauty | 0.6 | 1.1 | | 4.4/4.4/4.6 |
