# Supplementary material for: The rare orange-red colored Euphorbia pulcherrima cultivar ‘Harvest Orange’ shows a nonsense mutation in a flavonoid 3’-hydroxylase allele expressed in the bracts
Source: BMC Plant Biol. 2018 Oct 3;18:216. doi: 10.1186/s12870-018-1424-0 (PMC6171185; doi:10.1186/s12870-018-1424-0)
Supplement: Supplementary file 6 — Figure S2. Multiple alignment of the open reading frames of the F3′H cDNA clones of Euphorbia pulcherrima cvs. Harvest Orange (EpHO_F3′H, KY273441), Premium Red (EpPR_F3′H, KY489667), Christmas Beauty (EpCB_F3′H, KY273439), and Christmas Feelings (EpCF_F3′H, KY273440). (DOCX 23 kb) [file 12870_2018_1424_MOESM6_ESM.docx]

**Figure S2:** Multiple alignment of the open reading frames of the *F3'H* cDNA clones of *Euphorbia pulcherrima* cvs. Harvest Orange (*Ep*HO_*F3'H*, KY273441), Premium Red (*Ep*PR_*F3'H*, KY489667), Christmas Beauty (*Ep*CB_*F3'H*, KY273439), and Christmas Feelings (*Ep*CF_*F3'H*, KY273440).

1 50

EpHO_F3’H ATGTTACCAC TCTTTGCGTT TACCATTTTT TCTGCCATTT TTTTTGAAAA

EpPR_F3’H ATGTTACCAC TCTTTGCGTT TACCATTTTT TCTGCCATTT T.........

EpCB_F3’H ATGTTACCAC TCTTTGCGTT TACCATTTTT TCTGCCATTT T.........

EpCF_F3’H ATGTTACCAC TCTTTGCGTT TACCATTTTT TCTGCCATTT T.........

51 100

EpHO_F3’H CCATTTTTTC TGCCATTTTC ATTTCCTTTT TTTTCTTCTT CTTTTTTCGC

EpPR_F3’H .......... .........C ATTTCCTTTT T...CTTCTT CTTTTTTCGC

EpCB_F3’H .......... .........C ATTTCCTTTT T...CTTCTT CTTTTTTCGC

EpCF_F3’H .......... .........C ATTTCCTTTT TTTTCTTCTT CTTTTTTCGC

101 150

EpHO_F3’H CGTACCTCTC GCCCCCCTCT TCCTCCCGGT CCTAGACCAC TGCCTGTAAT

EpPR_F3’H CGTACCTCTC GCCCCCCTCT TCCTCCCGGT CCTAGACCAC TGCCTGTAAT

EpCB_F3’H CGTACCTCTC GCCCCCCTCT TCCTCCCGGT CCTAGACCAC TGCCTGTAAT

EpCF_F3’H CGTACCTCTC GCCCCCCTCT TCCTCCCGGT CCTAGACCAC TGCCTGTAAT

151 200

EpHO_F3’H TGGAAACCTG CCTCATTTAG GCCCCAAACC CCACCAGTCA ATAGCCTCCT

EpPR_F3’H TGGAAACCTG CCTCATTTAG GCCCCAAACC CCACCAGTCA ATAGCCTCCT

EpCB_F3’H TGGAAACCTG CCTCATTTAG GCCCCAAACC CCACCAGTCA ATAGCCTCCT

EpCF_F3’H TGGAAACCTG CCTCATTTAG GCCCCAAACC CCACCAGTCA ATAGCCTCCT

201 250

EpHO_F3’H TGGCTCGGGT TTATGGCCCC CTTATGCACC TCCGTATAGG CTTTGTCGAC

EpPR_F3’H TGGCTCGGGT TTATGGCCCC CTTATGCACC TCCGTATGGG CTTTGTCGAC

EpCB_F3’H TGGCTCGGGT TTATGGCCCC CTTATGCACC TCCGTATGGG CTTTGTCGAC

EpCF_F3’H TGGCTCGGGT TTATGGCCCC CTTATGCACC TCCGTATAGG CTTTGTCGAC

251 300

EpHO_F3’H GTCGTTGTGG CGGCGTCGGC GTCCGTTGCT GCCCAGTTCT TGAAAGCTCA

EpPR_F3’H GTCGTTGTGG CGGCGTCGGC GTCCGTTGCT GCCCAGTTCT TGAAAGCTCA

EpCB_F3’H GTCGTTGTGG CGGCGTCGGC GTCCGTTGCT GCCCAGTTCT TGAAAGCTCA

EpCF_F3’H GTCGTTGTGG CGGCGTCGGC GTCCGTTGCT GCCCAGTTCT TGAAAGCTCA

301 350

EpHO_F3’H TGACGCTAAT TTCTCGAGCC GGCCGCCTAA TTCGGGTGCT AAGTATGTTG

EpPR_F3’H TGACGCTAAT TTCTCGAGCC GGCCGCCTAA TTCGGGTGCT AAGTATGTTG

EpCB_F3’H TGACGCTAAT TTCTCGAGCC GGCCGCCTAA TTCGGGTGCT AAGTATGTTG

EpCF_F3’H TGACGCTAAT TTCTCGAGCC GGCCGCCTAA TTCGGGTGCT AAGTATGTTG

351 400

EpHO_F3’H CTTATAATTA CCAAGATCTT GTTTTTGCCC CGTACGGACC TCGCTGGCGC

EpPR_F3’H CTTATAATTA CCAAGATCTT GTTTTTGCCC CGTACGGACC TCGCTGGCGC

EpCB_F3’H CTTATAATTA CCAAGATCTT GTTTTTGCCC CGTACGGACC TCGCTGGCGC

EpCF_F3’H CTTATAATTA CCAAGATCTT GTTTTTGCCC CGTACGGACC TCGCTGGCGC

401 450

EpHO_F3’H ATGCTCAGGA AAATCAGTGC CGTGCATCTC TTCTCGGCTA AGGCCTTGGA

EpPR_F3’H ATGCTCAGGA AAATCAGTGC CGTGCATCTC TTCTCGGCTA AGGCCTTGGA

EpCB_F3’H ATGCTCAGGA AAATCAGTGC CGTGCATCTC TTCTCGGCTA AGGCCTTGGA

EpCF_F3’H ATGCTCAGGA AAATCAGTGC CGTGCATCTC TTCTCGGCTA AGGCCTTGGA

451 500

EpHO_F3’H TGATTTCCGC CATGTTAGAC AGGAAGAAGT GGCAATCCTT GTACGGTCTC

EpPR_F3’H TGATTTCCGC CATGTTAGAC AGGAAGAAGT GGCAATCCTT GTACGGTCTC

EpCB_F3’H TGATTTCCGC CATGTTAGAC AGGAAGAAGT GGCAATCCTT GTACGGTCTC

EpCF_F3’H TGATTTCCGC CATGTTAGAC AGGAAGAAGT GGCAATCCTT GTACGGTCTC

501 550

EpHO_F3’H TAGTAAGTTC CGGGCACGAA AGAGCGGTCA ATTTAGGGCA TCTGGTGAAC

EpPR_F3’H TAGTAAGTTC CGGGCACGAA AGAGCGGTCA ATTTAGGGCA TCTGGTGAAC

EpCB_F3’H TAGTAAGTTC CGGGCACGAA AGAGCGGTCA ATTTAGGGCA TCTGGTGAAC

EpCF_F3’H TAGTAAGTTC CGGGCACGAA AGAGCGGTCA ATTTAGGGCA TCTGGTGAAC

551 600

EpHO_F3’H CTGTGCGCCA CAAATGCACT GGCACGCGTA ATGATTGGCA GAAGAGTATT

EpPR_F3’H CTGTGCGCCA CAAATGCACT GGCACGCGTA ATGATTGGCA GAAGAGTATT

EpCB_F3’H CTGTGCGCCA CAAATGCACT GGCACGCGTA ATGATTGGCA GAAGAGTATT

EpCF_F3’H CTGTGCGCCA CAAATGCACT GGCACGCGTA ATGATTGGCA GAAGAGTATT

601 650

EpHO_F3’H CAGCGACAGC GGTGATCCGA AGGCCGACGA GTTCAAGTCA ATGGTGGTGG

EpPR_F3’H CAGCGACAGC GGTGATCCGA AGGCCGACGA GTTCAAGTCA ATGGTGGTGG

EpCB_F3’H CAGCGACAGC GGTGATCCGA AGGCCGACGA GTTCAAGTCA ATGGTGGTGG

EpCF_F3’H CAGCGACAGC GGTGATCCGA AGGCCGACGA GTTCAAGTCA ATGGTGGTGG

651 700

EpHO_F3’H AACTGATGAG ACCTGCCGGA GTATTCAATA TAGGGGATTT TATTCCGGCA

EpPR_F3’H AACTGATGAG ACTTGCCGGA GTATTCAATA TAGGGGATTT TATTCCGGCA

EpCB_F3’H AACTGATGAG ACTTGCCGGA GCATTCAATA TAGGGGATTT TATTCCGGCA

EpCF_F3’H AACTGATGAG ACTTGCCGGA GTATTCAATA TAGGGGATTT TATTCCGGCA

701 750

EpHO_F3’H CTGGAGTGGC TGGATTTACA GCGAGTAGCA GCTAAAATGA AGAAACTCCA

EpPR_F3’H CTGGAGTGGC TGGATTTACA GCGAGTAGCA GCTAAAATGA AGAAACTCCA

EpCB_F3’H CTGGAGTGGC TGGATTTACA GCGAGTAGCA GCTAAAATGA AGAAACTCCA

EpCF_F3’H CTGGAGTGGC TGGATTTACA GCGAGTAGCA GCTAAAATGA AGAAACTCCA

751 800

EpHO_F3’H TAAGAGATTC GATGCGTTTT TGACTGAAAT CGTCGAGGAA CACAAGAGTA

EpPR_F3’H TAAGAGATTC GATGCGTTTT TGACTGAAAT CGTCGAGGAA CACAAGAGTA

EpCB_F3’H TAAGAGATTC GATGCGTTTT TGACTGAAAT CGTCGAGGAA CACAAGAGTA

EpCF_F3’H TAAGAGATTC GATGCGTTTT TGACTGAAAT CGTCGAGGAA CACAAGAGTA

801 850

EpHO_F3’H ACAAAGGAGA GTCAACTCAC AGAGACATGT TGACTACTTT AATCTCGTTA

EpPR_F3’H ACAAAGGAGA GTCAACTCAC AGAGACATGT TGACTACTTT AATCTCGTTA

EpCB_F3’H ACAAAGGAGA GTCAACTCAC AGAGACATGT TGACTACTTT AATCTCGTTA

EpCF_F3’H ACAAAGGAGA GTCAACTCAC AGAGACATGT TGACTACTTT AATCTCGTTA

851 900

EpHO_F3’H AAGGAGGAAG AAGCTGATGA CGGTGAGGGA GGGAAAATCA CTGACACCGA

EpPR_F3’H AAGGAGGAAG AAGCTGATGA CGGTGAGGGA GGGAAAATCA CTGACACCGA

EpCB_F3’H AAGGAGGAAG AAGCTGATGA CGGTGAGGGA GGGAAAATCA CTGACACCGA

EpCF_F3’H AAGGAGGAAG AAGCTGATGA CGGTGAGGGA GGGAAAATCA CTGACACCGA

901 950

EpHO_F3’H AATTAAAGCC CTGCTTCTGA ACATGTTTGC AGCAGGCACC GACACTACAT

EpPR_F3’H AATTAAAGCC CTGCTTCTGA ACATGTTTGC AGCAGGCACC GACACTACAT

EpCB_F3’H AATTAAAGCC CTGCTTCTGA ACATGTTTGC AGCAGGCACC GACACTACAT

EpCF_F3’H AGTTAAAGCC CTGCTTCTGA ACATGTTTGC AGCAGGCACC GACACTACAT

951 1000

EpHO_F3’H CAAGCACGGT TGAGTGGGCC ATTGCTGAGC TCATCAGGCA CCCCAAAATA

EpPR_F3’H CCAGCACAGT TGAGTGGGCC ATTGCTGAGC TCATCAGGCA CCCCAAAATA

EpCB_F3’H CCAGCACAGT TGAGTGGGCC ATTGCTGAGC TCATCAGGCA CCCCAAAATA

EpCF_F3’H CAAGCACGGT TGAGTGGGCC ATTGCTGAGC TCATCAGGCA CCCCAAAATA

1001 1050

EpHO_F3’H CTAACCAAAC TCCGGCAAGA ACTCGACTCC GTCGTCGGCG CCGATTGTCT

EpPR_F3’H CTAACCAAAC TCCGGCGAGA ACTCGACTCC GTCGTCGGCG CCGATTGTCT

EpCB_F3’H CTAACCAAAC TCCGGCGAGA ACTCGACTCC GTCGTCGGCG CCGATTGTCT

EpCF_F3’H CTAACCAAAC TCCGGCAAGA ACTCGACTCC GTCGTCGGCG CCGATTGTCT

1051 1100

EpHO_F3’H CGTAACCGAG CTAGACATCA CTCAACTCCC CTACCTCCAA GCCGTCGTCA

EpPR_F3’H CGTAACCGAG CTAGACATCA CTCAACTCCC CTACCTCCAA GCCGTCGTCA

EpCB_F3’H CGTAACCGAG CTAGACATCA CTCAACTCCC CTACCTCCAA GCCGTCGTCA

EpCF_F3’H CGTAACCGAG CTAGACATCA CTCAACTCCC CTACCTCCAA GCCGTCGTCA

1101 1150

EpHO_F3’H AAGAAACCTT CCGCCTCCAC CCATCAACTC CCCTCTCTCT CCCTCGAATG

EpPR_F3’H AAGAAACCTT CCGCCTCCAC CCATCAACTC CCCTCTCTCT CCCTCGAATG

EpCB_F3’H AAGAAACCTT CCGCCTCCAC CCATCAACTC CCCTCTCTCT CCCTCGAATG

EpCF_F3’H AAGAAACCTT CCGCCTCCAC CCATCAACTC CCCTCTCTCT CCCTCGAATG

1151 1200

EpHO_F3’H GCGGCCGAAA GCTGCGAAAT CAACGGCTAC CACATCCCAA AAGGCGCCAC

EpPR_F3’H GCGGCCGAAA GCTGCGAAAT CAACGGCTAC CACATCCCAA AAGGCGCCAC

EpCB_F3’H GCGGCCGAAA GCTGCGAAAT CAACGGCTAC CACATCCCAA AAGGCGCCAC

EpCF_F3’H GCGGCCGAAA GCTGCGAAAT CAACGGCTAC CACATCCCAA AAGGCGCCAC

1201 1250

EpHO_F3’H GCTTCTGGTC AACGTGTGGG CAATAACTCG CGATCCAGAA GTATGGAAAG

EpPR_F3’H GCTTCTGGTC AACGTGTGGG CAATAGCTCG CGATCCAGAA GTATGGAAAG

EpCB_F3’H GCTTCTGGTC AACGTGTGGG CAATAGCTCG CGATCCAGAA GTATGGAAAG

EpCF_F3’H GCTTCTGGTC AACGTGTGGG CAATAGCTCG CGATCCAGAA GTATGGAAAG

1251 1300

EpHO_F3’H AGCCGCTGGA GTTTCGACCG GAGAGGTTTC TCGCCGGCGG AGAAAGGCCG

EpPR_F3’H AGCCGCTGGA GTTTCGACCG GAGAGGTTTC TCGCCGGCGG AGAAAGGCCG

EpCB_F3’H AGCCGCTGGA GTTTCGACCG GAGAGGTTTC TCGCCGGCGG AGAAAGGCCG

EpCF_F3’H AGCCGCTGGA GTTTCGACCG GAGAGGTTTC TCGCCGGCGG AGAAAGGCCG

1301 1350

EpHO_F3’H AACGCCGACG TGAAAGGGAC GGATTTTGAG GTGATTCCGT TTGGGGCAGG

EpPR_F3’H AACGCCGACG TGAAAGGGAC GGATTTTGAG GTGATTCCGT TTGGGGCAGG

EpCB_F3’H AACGCCGACG TGAAAGGGAC GGATTTTGAG GTGATTCCGT TTGGGGCAGG

EpCF_F3’H AACGCCGACG TGAAAGGGAC GGATTTTGAG GTGATTCCGT TTGGGGCAGG

1351 1400

EpHO_F3’H GCGGAGAATT TGCGCAGGGA TGAATTTAGG GTTAGTAATG GTTCATCTGC

EpPR_F3’H GCGGAGAATT TGCGCAGGGA TGAATTTAGG GTTAGTAATG GTTCAGCTGC

EpCB_F3’H GCGGAGAATT TGCGCAGGGA TGAATTTAGG GTTAGTAATG GTTCAGCTGC

EpCF_F3’H GCGGAGAATT TGCGCAGGGA TGAATTTAGG GTTAGTAATG GTTCATCTGC

1401 1450

EpHO_F3’H TTATTGCGAG TTTAGTACAA GGATTTGAAT GGGAATTGGA AAGGGAAAAA

EpPR_F3’H TTATTGCGAG TTTAGTACAA GGATTTGAAT GGGAATTGGA AAGGGAAAAA

EpCB_F3’H TTATTGCGAG TTTAGTACAA GGATTTGAAT GGGAATTGGA AAGGGAAAAA

EpCF_F3’H TTATTGCGAG TTTAGTACAA GGATTTGAAT GGGAATTGGA AAGGGAAAAA

1451 1500

EpHO_F3’H CCAGAGAAAT TGAACATGGA GGAAGCTTAT GGGCTGACCT TACAACGACT

EpPR_F3’H CCAGAGAAAT TGAACATGGA GGAAGCTTAT GGGCTGACCT TACAACGACT

EpCB_F3’H CCAGAGAAAT TGAACATGGA GGAAGCTTAT GGGCTGACCT TACAACGACT

EpCF_F3’H CCAGAGAAAT TGAACATGGA GGAAGCTTAT GGGCTGACCT TACAACGACT

1501 1550

EpHO_F3’H TGAGCCATTG ATGGTGTACC CAAACCCTAG GTTGTCGTCT CTAGTCTATG

EpPR_F3’H TGAGCCATTG ATGGTGCACC CAAACCCTAG GTTGTCGTCT CTAGTCTATG

EpCB_F3’H TGAGCCATTG ATGGTGCACC CAAACCCTAG GTTGTCGTCT CTAGTCTATG

EpCF_F3’H TGAGCCATTG ATGGTGTACC CAAACCCTAG GTTGTCGTCT CTAGTCTATG

1551 1564

EpHO_F3’H CTGCTCCTAT TTGA

EpPR_F3’H CTGCGCCTGT TTGA

EpCB_F3’H CTGCGCCTGT TTGA

EpCF_F3’H CTGCTCCTGT TTGA
