# Supplementary material for: The rare orange-red colored Euphorbia pulcherrima cultivar ‘Harvest Orange’ shows a nonsense mutation in a flavonoid 3’-hydroxylase allele expressed in the bracts
Source: BMC Plant Biol. 2018 Oct 3;18:216. doi: 10.1186/s12870-018-1424-0 (PMC6171185; doi:10.1186/s12870-018-1424-0)
Supplement: Supplementary file 7 — Figure S3. Phylogenetic analysis of F3′Hs from the three poinsettia cvs. Christmas Feelings (KY273440), Christmas Beauty (KY273439) and Premium Red (KY489667) by application of the Maximum Likehood method based on the deduced amino acid sequences of isolated poinsettia F3′H cDNA clones and deduced F3′H amino acid sequences of other species available in the NCBI database. Sequences of FNSII were used as outgroup. The JJT matrix-based model was used as a substitution model. The percentage of trees in which the associated taxa clustered together is shown next to the branches. (DOCX 20 kb) [file 12870_2018_1424_MOESM7_ESM.docx]

**Figure S3:** Phylogenetic analysis of F3’Hs from the three poinsettia cvs. Christmas Feelings (KY273440), Christmas Beauty (KY273439) and Premium Red (KY489667) by application of the Maximum Likehood method based on the deduced amino acid sequences of isolated poinsettia F3’H cDNA clones and deduced F3’H amino acid sequences of other species available in the NCBI database. Sequences of FNSII were used as outgroup. The JJT matrix-based model was used as a substitution model. The percentage of trees in which the associated taxa clustered together is shown next to the branches.
