# Supplementary material for: The rare orange-red colored Euphorbia pulcherrima cultivar ‘Harvest Orange’ shows a nonsense mutation in a flavonoid 3’-hydroxylase allele expressed in the bracts
Source: BMC Plant Biol. 2018 Oct 3;18:216. doi: 10.1186/s12870-018-1424-0 (PMC6171185; doi:10.1186/s12870-018-1424-0)
Supplement: Supplementary file 8 — Figure S4. Multiple Alignment of the deduced amino acid sequences of the DFRs of Euphorbia pulcherrima cvs. Harvest Orange (EpHO_DFR, KY273438), Premium Red (EpPR_DFR, KY499617), Christmas Beauty (EpCB_DFR, KY273436), and Christmas Feelings (EpCF_DFR, KY273437). Grey frames highlight the VDV region in position 132 to 134. Grey shades highlight differences in the amino acid sequence. (DOCX 21 kb) [file 12870_2018_1424_MOESM8_ESM.docx]

**Figure S4:** Multiple Alignment of the deduced amino acid sequences of the DFRs of *Euphorbia pulcherrima* cvs. Harvest Orange (*Ep*HO_DFR, KY273438), Premium Red (*Ep*PR_DFR, KY499617), Christmas Beauty (*Ep*CB_DFR, KY273436), and Christmas Feelings (*Ep*CF_DFR, KY273437). Grey frames highlight the VDV region in position 132 to 134. Grey shades highlight differences in the amino acid sequence.

1 50

EpHO_DFR MGEVPEIVCV TGASGFIGSW LIMRLLERGY RVRATVRDPG NISKVQHLIE

EpPR_DFR MGEVPEIVCV TGASGFIGSW LIMRLLERGY RVRATVRDPG NISKVQHLIE

EpCB_DFR MGEVPEIVCV TGASGFIGSW LIMRLLERGY RVRATVRDPG NISKVQHLIE

EpCF_DFR MGEVPEIVCV TGASGFIGSW LIMRLLERGY RVRATVRDPG NISKVQHLIE

51 100

EpHO_DFR LPNAMTNLSL WKADLSVEGS FDEAIKGCSG VFHVATPMDF DSKDPENEVI

EpPR_DFR LPNAKTNLSL WKADLSVEGS FDEAIKGCSG VFHVATPMDF DSKDPENEVI

EpCB_DFR LPNAMTNLSL WKADLSVEGS FDEAIKGCSG VFHVATPMDF DSKDPENEVI

EpCF_DFR LPNAKTNLSL WKADLSVEGS FDEAIKGCSG VFHVATPMDF DSKDPENEVI

101 150

EpHO_DFR KPTVSGVLDI MKACSKAKTV RRIIFTSSAG TVDVEQHKKP LYDESCWSDL

EpPR_DFR KPTVSGVLDI MKACSKAKTV RRIIFTSSAG TVDVEQHKKP LYDESCWSDL

EpCB_DFR KPTVSGVLDI MKACSKAKTV RRIIFTSSAG TVDVEQHKKP LYDESCWSDL

EpCF_DFR KPTVRGVLDI MKACSKAKTV RRIIFTSSAG TVDVEQHKKP LYDENCWSDL

151 200

EpHO_DFR DFILSTKMTG WMYFVSKTMA EKAAWKYAEE NNIDLISIIP TLVVGPFIMP

EpPR_DFR DFILATKMTG WMYFVSKTMA EKAAWKFAEE NNIDLISIIP TLVVGPFIMP

EpCB_DFR DFILATKMTG WTYFVSKTMA EKAAWKFAEE NNIDLISIIP TLVVGPFIMP

EpCF_DFR DFILATKMTG WMYFVSKTMA EKAAWKFAEE NNIDLISIIP TLVVGPFIMP

201 250

EpHO_DFR SMPPSLITAL SPITGNEAHY SIIKQGHYIH LDDLCNAHIY LFEHSKAKGR

EpPR_DFR SMPPSLITAL SPITGNEAHY SIIKQGHYIH LDDLCNAHIY LFEHSKAKGR

EpCB_DFR SMPPSLITAL SPITGNEAHY SIIKQGHYIH LDDLCNAHIY LFEHSKAKGR

EpCF_DFR SMPPSLITAL SPITGNEAHY SIIKQGHYIH LDDLCNAHIY LFEHSKAKGR

251 300

EpHO_DFR YFCSSHDATI HEIARLLRQK YPQFNIPAKI KGVEENVKNL IFSSKKLEEA

EpPR_DFR YFCSSHDATI HEIARLLRQK YPQFNIPTKI KGVEENVKNL IFSSKKLEEA

EpCB_DFR YFCSSHDATI HEIARLLRQK YPQFNIPTKI KGVEENVKNL IFSSKKLEEA

EpCF_DFR YFCSSHDATI HEIARLLRQK YPQFNIPTKI KGVEENVKNL IFSSKKLEEA

301 352

EpHO_DFR GFEFKYSLED MFEGAVETCL AKGLLHAADE KQEPNKVETN DVITSTAEVSCH

EpPR_DFR GFEFKYSLED MFEGAVETCL AKGLLHAADE KQEPNKVETN DVITSTAEVSCG

EpCB_DFR GFEFKYSLED MFEGAVETCL AKGLLHAADE KQEPNKVETN DVITSTAEVSCG

EpCF_DFR GFEFKYSLED MFEGAVETCL AKGLLHAADE KQEPNKVETN DVITSTAEVSCG
